# Design and research of music teaching system based on virtual reality system in the context of education informatization

**DOI:** 10.1371/journal.pone.0285331

**Published:** 2023-10-05

**Authors:** Yan Feng

**Affiliations:** School of Medical Humanities, Xinxiang Medical University, Xinxiang, 453000, China; Vellore Institute of Technology: VIT University, INDIA

## Abstract

Virtual Reality (VR) technology uses computers to simulate the real world comprehensively. VR has been widely used in college teaching and has a huge application prospect. To better apply computer-aided instruction technology in music teaching, a music teaching system based on VR technology is proposed. First, a virtual piano is developed using the HTC Vive kit and the Leap Motion sensor fixed on the helmet as the hardware platform, and using Unity3D, related SteamVR plug-ins, and Leap Motion plug-ins as software platforms. Then, a gesture recognition algorithm is proposed and implemented. Specifically, the Dual Channel Convolutional Neural Network (DCCNN) is adopted to collect the user’s gesture command data. The dual-size convolution kernel is applied to extract the feature information in the image and the gesture command in the video, and then the DCCNN recognizes it. After the spatial and temporal information is extracted, Red-Green-Blue (RGB) color pattern images and optical flow images are input into the DCCNN. The prediction results are merged to obtain the final recognition result. The experimental results reveal that the recognition accuracy of DCCNN for the Curwen gesture is as high as 96%, and the recognition accuracy varies with different convolution kernels. By comparison, it is found that the recognition effect of DCCNN is affected by the size of the convolution kernel. Combining convolution kernels of size 5×5 and 7×7 can improve the recognition accuracy to 98%. The research results of this study can be used for music teaching piano and other VR products, with extensive popularization and application value.

## Introduction

With the rapid growth of science and lifestyle change, the way of education and learning is also facing the challenge and opportunity of significant transformation and development. The coronavirus disease 2019 (COVID-19) outbreak has provided a broad space for the development of online music education, but there are many problems with online music teaching [1,2]. Therefore, the study of online music education models with remarkable learning effects and rich learning content has become one of the issues of common concern in society. The goal of music teaching is to cultivate musical talents who have well musical accomplishments and are applied to the teaching tasks of middle and primary schools. The training process is intensely technical [3,4]. Virtual Reality (VR) is a kind of simulation technology that uses computers, sensors, and various auxiliary devices to create a virtual world to reproduce the real world as much as possible. The most typical feature of VR is immersion, which affects users through sight, hearing, and touch to make them feel immersive. Using VR technology to create realistic virtual scenes and environments is often much cheaper and less difficult than building the real world. Therefore, it has a wide application prospect in many fields such as military training, experimental teaching, industrial design, architectural display, medical anatomy, and virtual games. VR technology has four basic characteristics: existence, interactivity, creativity, and multi-perception. Existence refers to the stereoscopic and verisimilitude of the 3D image scene through computer simulation. Interactivity means the natural interaction between humans and machines, operating things in the virtual environment through a mouse and keyboard or with the help of sensing devices. Creativity refers to creating new vivid physical landscapes such as the water flow in the virtual ocean world according to the laws of physical motion. Multi-perception indicates the use of visual, auditory, and tactile devices to make VR systems multi-perceptual.

At present, there are still many deficiencies in the music teaching system in schools, which can be divided into the following two aspects. 1. The use of modern teaching equipment and the corresponding teaching tools. Music classroom teaching enables students to understand music and through the audiovisual combination to learn music through sound. With the progress of technology and the country’s emphasis on education, multimedia devices have gradually entered the classroom of every school. Although multimedia facilities are available in some schools, they are not used to their fullest extent. Teachers need to use multimedia equipment to promote the classroom teaching of music and stimulate students’ interest in learning music by projecting pictures, videos, and recordings on multimedia. Students can learn about some performers’ classic works and so on. At the same time, to enable students to get closer to and understand music, the school also needs to be equipped with corresponding teaching tools, such as piano, electronic keyboard, cucurbit flute, flute, and other musical instruments. The purpose is to enable students to learn musical instruments in music lessons, learn music theory knowledge, and improve their musical literacy. 2. Innovation of teaching mode and teaching concept. Nowadays, to effectively improve the quality of music teaching in school and make students have good music literacy through music teaching, firstly, the traditional teaching mode and teaching concept should be completely changed. Combining with the requirements of the new curriculum standards, the quality education goal is taken as the guiding direction of students’ music teaching mode and concept. The "indoctrination" teaching method in the traditional teaching mode should be transformed, highlighting the main position of students in learning, actively communicating with students, and guiding them to fully realize the importance of music learning to their healthy growth.

In this context, this study developed a virtual piano with an HTC Vive kit and Leap Motion sensor fixed on the helmet as the hardware platform, and Unity3D and related SteamVR plug-in and Leap Motion plug-in as the software platform. The virtual piano is composed of a virtual keyboard with Cube components. The script response function of being approached and pressed or released by Cube components is compiled to record the performance events of the virtual piano keyboard. The sound of the virtual piano is further realized with the help of the Musical Instrument Digital Interface (MIDI). To enhance the performance, a gesture recognition algorithm is proposed and implemented using machine learning (ML) theory. Specifically, Dual Channel Convolutional Neural Network (DCCNN) is used to collect the user’s gesture command data. The dual-size convolution kernel is employed to extract the feature information in the image and the gesture instruction in the video. Then the DCCNN recognizes it. After the spatial and temporal information is extracted, color pattern images and optical flow images of the Red-Green-Blue (RGB) are fed into the DCCNN, and the prediction results are merged to get the final recognition result. The gesture recognition algorithm implemented in this study can be used not only for the property setting of virtual piano but also for other VR products, which have a wide range of promotion and application value.

## Literature review

In optimizing the music teaching system, many researchers have conducted in-depth explorations. Loveridge (2020) [5] pointed out that VR technology is a high-tech simulation technology active in many fields such as medicine, aerospace, industry, and games in recent years. VR technology can help students create conditions for lifelong learning through constantly changing novel environments and practical experiences, and improve students’ learning interest and learning effect. According to the relevant domestic and international research results of the application of VR technology in music teaching, based on the actual development of VR tools, they used literature analysis, case analysis, and questionnaire to investigate and analyze the use of VR technology in violin teaching, and to explore the limitations and prospects of this technology in music teaching. Soliman et al. (2021) [6] pointed out that due to the particularity of music teaching methods (combination of sensibility and rationality), the existing computer-assisted instruction technology is difficult to be applied in music teaching. This study proposed a VR technology-based music teaching system, which was based on audio processing analysis and brings unique immersion, interactivity, and conceivability through VR technology. This system provided a new model for music teaching preparation, process, and interaction. Gong (2021) [7] proposed a VR-based music teaching and training system. The system includes an instrument audio management module, audio waveform simulation module, instrument type judgment module, and training instrument error correction module, in which the instrument audio management module is employed to transmit the audio signal of the instrument; The simulation module is used to simulate the audio waveform data of the transmitted audio signal. The third module is adopted to automatically judge the instrument type according to the simulated audio waveform. When playing and training a certain instrument, the system will collect the playing audio of the instrument, voluntarily judge the instrument type of the audio, and automatically correct the instrument’s tone when playing a certain piece of music, thus improving the correction management during music training and performance. Xu et al. (2022) [8] pointed out that with the continuous reform and innovation of teaching methods in colleges, the curriculum system of students is also constantly enriched and developed. Hence, people’s requirements for teaching management and system are increasing. Physical education courses are usually outdoor teaching. Some schools have not established a complete teaching system. To this end, they designed an interactive sports teaching system based on artificial intelligence. Firstly, the hardware of the intelligent sports training interactive system was designed by constructing the total control circuit of the interactive teaching system, determining the corresponding circuit address decoding, improving the audio control circuit, and associating the video with the interactive driver. Secondly, the software design of this interactive system was carried out by creating an intelligent training function module, the design of a training database, and the realization of effective training and teaching of intelligent sports. Finally, the system test verified the corresponding effect, and the relevant system was further improved. Hua et al. (2022) [9] designed an intelligent auxiliary system to enhance the input accuracy and response speed of music training. The architecture is divided into the infrastructure, data, application, and presentation layers. In hardware design, the combination of an Advanced RISC Machine (ARM) and a digital signal processor (DSP) is used to realize data analysis, human-computer interaction (HCI), and interface interaction. In the software design, the Cepstrum algorithm is utilized to extract cepstrum features of music signals, and the linear smoothing algorithm is used for filtering. The dynamic time normalization method is adopted for pattern matching, and the radial basis function algorithm is employed to output results. Gong (2022) [10] argued that the traditional teaching mode of ethnic vocal music in colleges has such problems as low teaching quality, poor teaching diversity, and low interest of students. Based on this, they studied the innovation of a national vocal music teaching model in colleges based on a Deep Recurrent Neural Network (DRNN) algorithm. They designed a teaching quality evaluation model based on this algorithm. Data and information are collected from students’ class status, vocal music test results, classroom interaction, and other aspects. Moreover, the DRNN algorithm is used for comprehensive analysis to realize the multi-analysis and objective evaluation of the whole process of different teaching modes of national vocal music in music colleges.

It can be seen that with the development of digital image technology, the gradual popularization of three-dimensional (3D) panorama is the breakthrough point, and image-based VR technology gradually stands out. 3D panorama has attracted more and more attention due to its low development cost, strong realism, and convenient and quick panorama generation. However, the independent panorama technology lacks audio, so the teaching materials combined with Unity3D will make students feel the real audio-visual world.

## VR piano design based on UNITY 3D

### The construction of a VR hardware platform

**Fig 1:**
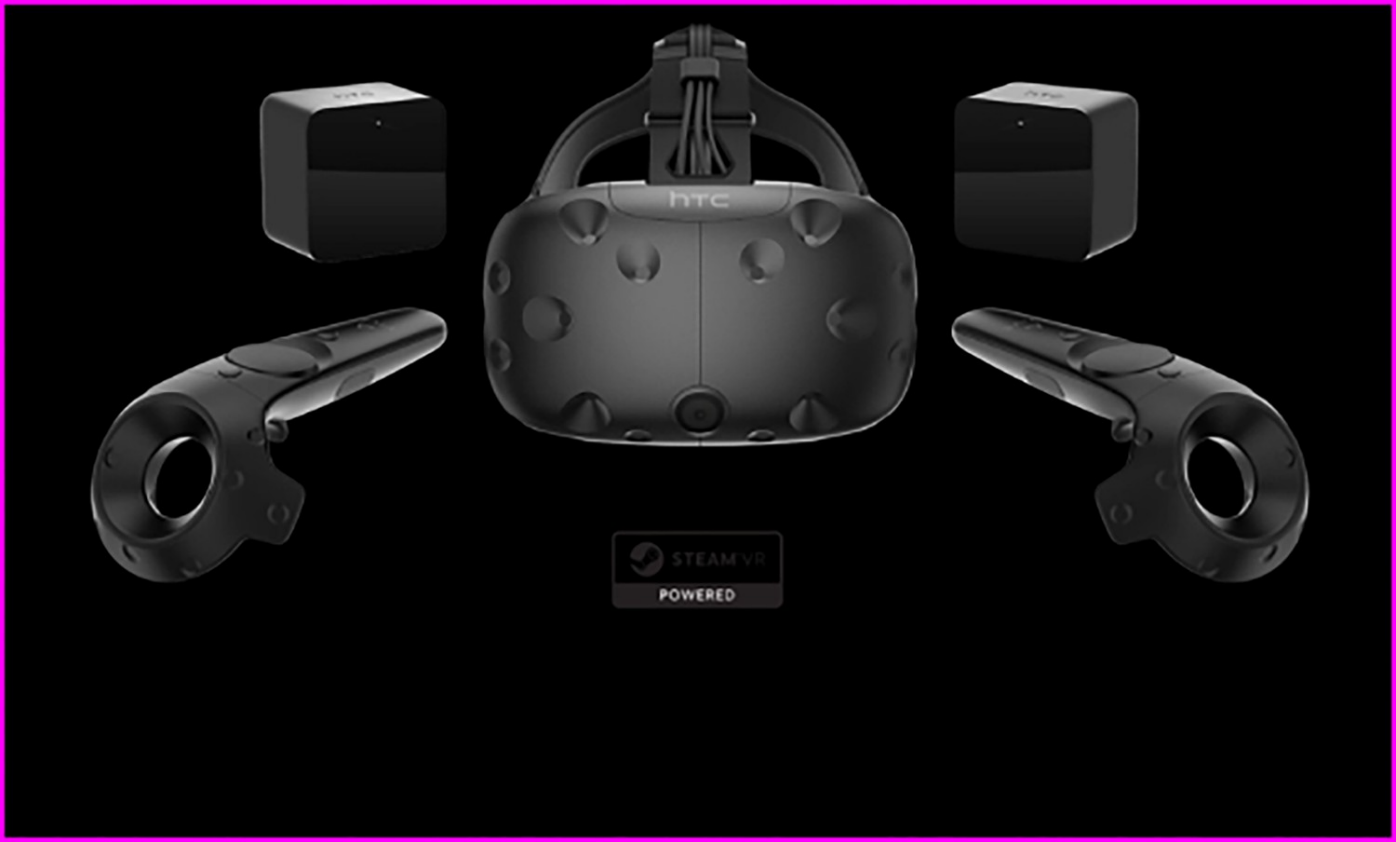
HTC Vive VR devices.

In this study, the HTC Vive VR kit is adopted as the hardware platform of the virtual piano. A complete set of HTC Vive VR devices is displayed in Fig 1, consisting of a helmet, two handles, and two positioners [11–13].

The Vive uses Lighting House indoor location technology. Two rows of Light Emitting Diodes (LEDs) inside the locator emit a scanning beam six times per second that alternately scans a location space with 15*15 feet in horizontal and vertical directions. The HTC Vive has over 70 photosensitive sensors on the helmet and handles [14,15]. As the beam sweeps through, the helmet begins to count. Once the sensor receives the scanning beam, it uses the relationship between the sensor position and the time it takes to receive the laser to calculate the exact position relative to the locator. With the help of location technology, users can walk around the virtual piano within the detection range and even play the piano on the other side of the keyboard, further increasing the sense of three-dimensionality and immersion. Here, the HTC Vive helmet and Leap Motion sensor are connected to computers through High Definition Multimedia Interface (HDMI) and Universal Serial Bus (USB) interfaces, respectively [16]. Table 1 lists the configuration properties of the computer and the built-in discrete graphics card used in this study.

**Table 1:** Computer configuration for virtual piano.

Graphics Processing Unit (GPU)	NVIDIA GeForce GTX 1060
Central Processing Unit (CPU)	Intel (R) Xeon (R) E5-2630
Random Access Memory (RAM)	16.0GB
Video output	16.0GB
USB terminal	USB3.0
Operating system	Windows 7

### The construction of the development platform of the VR piano software and the design of the virtual piano keyboard

Unity3D is a professional game engine developed by Unity Technologies, which enables game developers to easily complete various game creativity and 3D interactive development [17,18]. Unity3D has rendering resources such as physical simulation, normal mapping, screen space environment shading, dynamic shadow, etc. It has two major advantages over other game development tools: an extremely visual workflow and multi-dimensional cross-platform support. By the meaning of visual workflow, scene layout editing, resource binding, and the interactive object’s scripting language coding can be easily done. Unity3D spans multiple platforms in terms of deployment targets and can run on 21 platforms, including the current Windows, Mac, Wii, iPhone, WebGL, Windows, and Android [19,20].

The piano comprises 88 black and white keys, black and white keys separated by half a note, from left to right in the bass, middle and high registers. For simplicity, seven notes, do, re, mi, fa, so, la, and si, are set respectively in the bass, middle, and high registers of the developed virtual piano, with 21 white keys and 15 black chromatic keys. Canvas [21–23] is used in the piano interface of Unity3D, which carries the region of all User Interface (UI) elements. This component is bound to the piano key object in the Hierarchy panel, as exhibited in Table 2. There are three rendering modes for Canvas: Screen Space-overlay, World Space, and Screen Space-camera. In Screen space-overlay mode, the canvas fills the entire screen space and puts all UI elements under the canvas on the top layer of the screen, which is not blocked by any objects and can be seen without cameras. World Space is the world space mode. In this mode, the Canvas is located in the game scene like a 3D object, and its position and size can be set.

**Table 2:** Rendering modes for Canvas.

Rendering modes	Whether the canvas corresponds to the screen	Pixel correspondence	Cameras
Screen Space-Overlay	Yes	Optional	Not required
Screen Space-Camera	Yes	Optional	Need
World Space	No	Not optional	Need

## MIDI-based virtual piano sound effect and MIDI document management

### Analysis of the working principle of MIDI

During the performance, the MIDI keyboard sends the information by pressing the key to the computer in real-time, and the computer records the info individually. After the music is played, the MIDI format document is kept [24–26]. During replay, the computer interprets the key information one by one from the open MIDI document. With the help of pre-recorded acoustic files of each key or physical modeling, the key sound is synthesized accurately and timely according to each key’s playing time and strength, to realize the playback of the whole music. Based on the above basic working principle of MIDI, it is easy to understand that MIDI documents are equivalent to music scores and mainly consist of a series of keystroke events, called MIDI events. According to the MIDI specification, each MIDI Event consists of four parameters, the first parameter is a timestamp, that is, the time interval between the occurrence time of each event and the previous event; The second parameter is the name of the event, such as the press or release of a key; The third parameter is the key name, namely, which key is pressed or released; The fourth parameter is force, that is, the playing force of the key [27–29], which is generally expressed by speed. Table 3 presents the command format of the MIDI Event. In Table 3, the press or loosening of the key is denoted by 9x and 8x, respectively, and the key name and force are expressed by 1 byte, ranging from 1 to 128. Table 3 indicates that in addition to the Note On and Note Off events, there are many other events, such as Aftertouch. Since the notes being played change with time and force [30], using the event needs to be refined according to the length and force of the stay on the keyboard. Besides, the controller is an editor that can be used to control glissando, vibrato, and crescendo in MIDI instruments.

**Table 3:** MIDI event.

Category		Parameter (hexadecimal)
Bytes	Implication	
8x	Note off	Note (00-7F); Loosened note; Force: 00-7F
9x	Note on	Note (00-7F); The note pressed; Force: 00-7F
Ax	Key after Touch	Note: 00-7F; Force:00-7F
Bx	Controller	Controller number: 00-7F; Controller parameters: 00-7F
Cx	Program changes	Instrument No.: 00-7F
Dx	Aftertouch	Status: 0xA0-0xAF; Data: nnff
Ex	Portamento	Pitch low: pitch mod 128; Pitch high: pitch div 128
F0	System code	Total system code bytes: length variable; System code: Does not include the beginning F0, but includes the ending F7
FF	Other formats	Type of format: 00-FF
00-7F	Parameters of the last activated format (8x, 9x, Ax, Bx, Cx, Dx, Ex)	

### Property change settings for virtual piano

**Fig 2:**
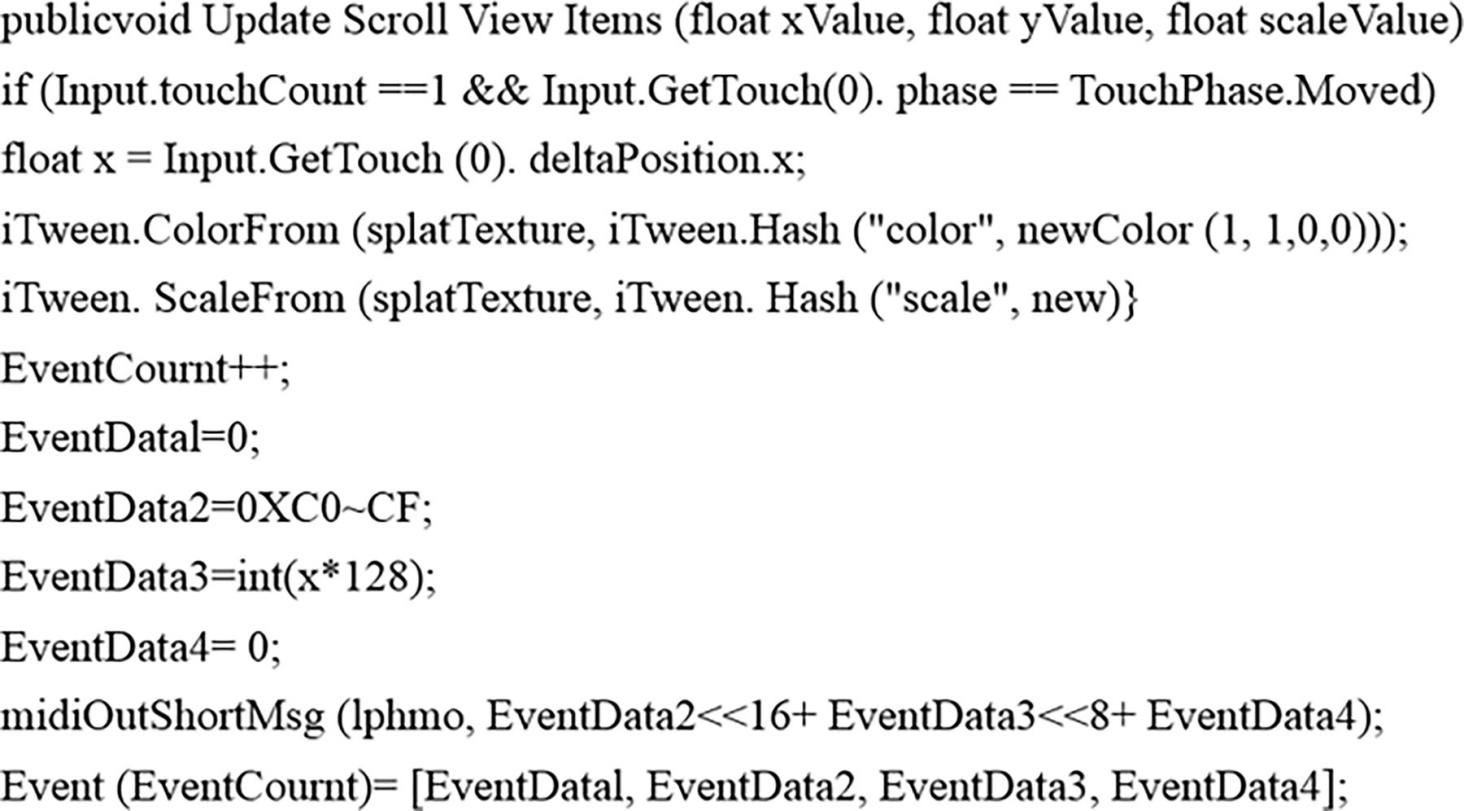
Code for property change settings.

In Table 3, many MIDI events can change the performance effect in addition to Note On and Note Off events. For example, changing the instrument type can change the timbre. The impact of the instrumental ensemble can be produced by assigning different channels of MIDI to various timbres. In this case, the channel function of MIDI must be used [31–33]. There are 16 channels in total. Additionally, changing the offset value of the keys can change the voice register; the tempo can change the playing speed, and so on. Because the mouse and keyboard cannot be used, it is even inconvenient to use the control handle in the virtual piano implemented here, so it is tough to change the property settings of the virtual piano in real-time. For this reason, this study uses gesture recognition to realize the property change settings of the virtual piano. The principle of gesture recognition will be introduced in detail in the next section. In Fig 2, a sliding bar is set on the upper left of the virtual piano keyboard. Gestures can control the sliding bar’s position and color to change the piano’s volume and select the instrument’s timbre.

### MIDI file archiving for music performance

Each key-playing event of the virtual piano and its property-changing event are recorded in the programmed array. The data recorded in the array is saved to a hard disk file. A MIDI file header is added to the file according to the MIDI specification, which can realize the MIDI file archive of the music played by the virtual piano. MIDI files usually start with a header block followed by one or more track blocks, with header blocks marked by ASCII code MThd and track blocks marked by ASCII code MTrk. The specific composition of the header block of a MIDI file contains information such as header type, header length, and data section. The header length is a variable constant, and the highest bit of each byte is the flag bit. The data section involves format, track number, partition, and a total of 3 16-bit bytes, which need to store high; The track block consists of a series of data streams made up of MIDI messages that are actually repositories of file song content data.

Syntax and format for header blocks:

<Header type><Header length><Format><Number of tracks><Partition>

<4d546864><00000006><ffff><nnnn><dddd>

The ID of the header block and track block is 0x4D546864 and 0x4D54726B; "00000006" is the number of bytes describing the length information of the header block. Currently, in MIDI protocol, this value is fixed at 6. "ffff" indicates a track format, which is generally a synchronous multi-track format; "dddd" specifies the basic time format type, which can be of two types. Type 1 defines the tick number of a quarter note, the tick being the smallest unit of time in MIDI. Type 2 defines the number of Simple Mail Transfer Protocol (SMTPE) frames per second and the tick of each SMTPE frame.

## Gesture recognition algorithm

### Gesture command data acquisition

**Fig 3:**
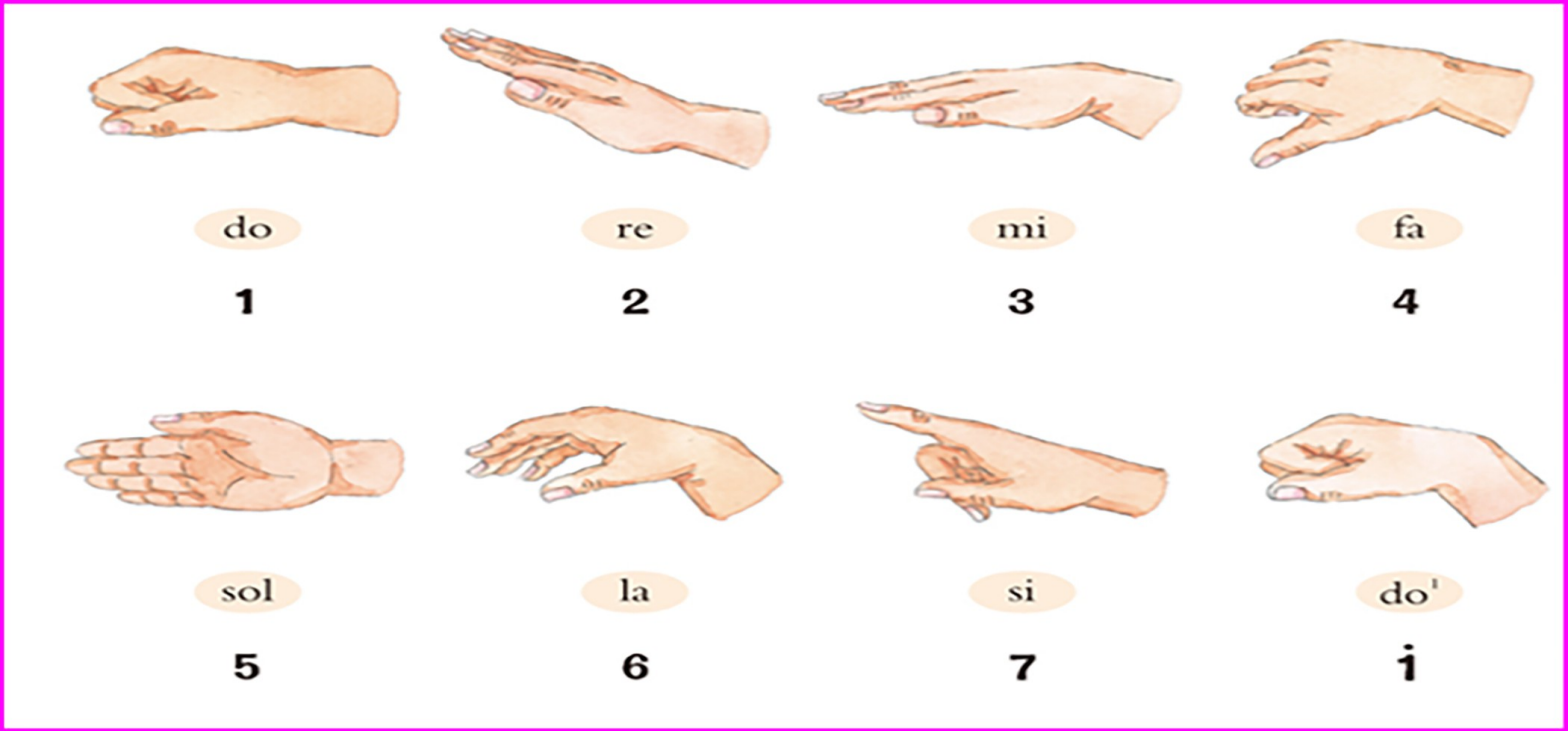
Curwen gesture.

Solfeggio is the skill of reading music scores using visual, auditory, and other perceptual methods. Solfeggio is helpful for students to quickly master the melody of music, enhance their perception and understanding of music, and improve their performance level. Rapid reflexes from vision to singing are required in this process, which requires intensive training. Some researchers use Convolutional Neural Networks (CNNs) combined with video tracking technology to identify the object. However, how to show the dynamic rhythm of singing and make the teaching process more straightforward and effective remains an open question. Curwen gesture by Hungarian music educator John. Curwen was created and used in music teaching. Thus, applying Curwen gesture to music perception teaching can increase learning interest, improve intonation, and enrich learning content. The main research content of this study is how to use the computer to recognize gestures in music teaching and establish the HCI music perception teaching method. The Curwen gesture is demonstrated in Fig 3.

**Fig 4:**
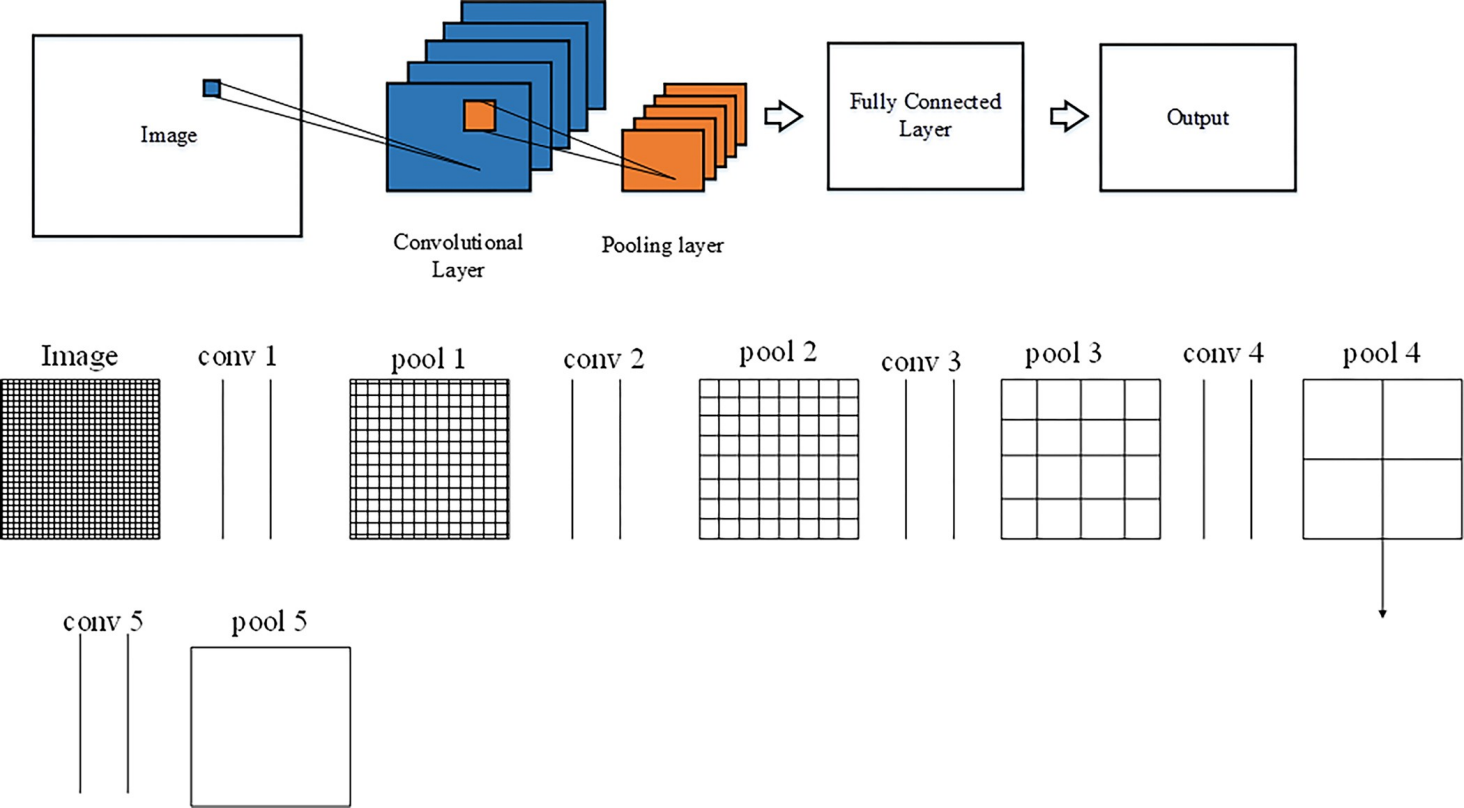
CNN structure.

CNN is a feedforward neural network that includes convolution computation. It can perform supervised and unsupervised learning by mimicking the visual mechanism of the human eye. The convolution kernel parameters in the hidden layer have the characteristics of weight sharing and interlayer connection, which makes it possible to learn the features of grid-like topology with reduced computation. As a result, CNN is often used to process images and speech. The structure of CNN consists of input, hidden, and output layers. The input layer can handle multidimensional data and must standardize input characteristics. The output layer can use logical or normalized functions to output the category tags. The hidden layer usually contains three common layers: convolutional layer, pooling layer, and fully connected (FC) layer, among which the convolutional and pooling layers are the unique structure of CNN. The convolutional layer contains the weight coefficient, while the pooling layer does not. The specific CNN structure is shown in Fig 4.

DCCNN is employed to recognize static gestures and extract advanced features to realize advanced recognition of multiple gestures, avoiding the preprocessing of traditional gesture recognition. DCCNN can obtain advanced features of different granularity and enhance gesture recognition.

**Fig 5:**
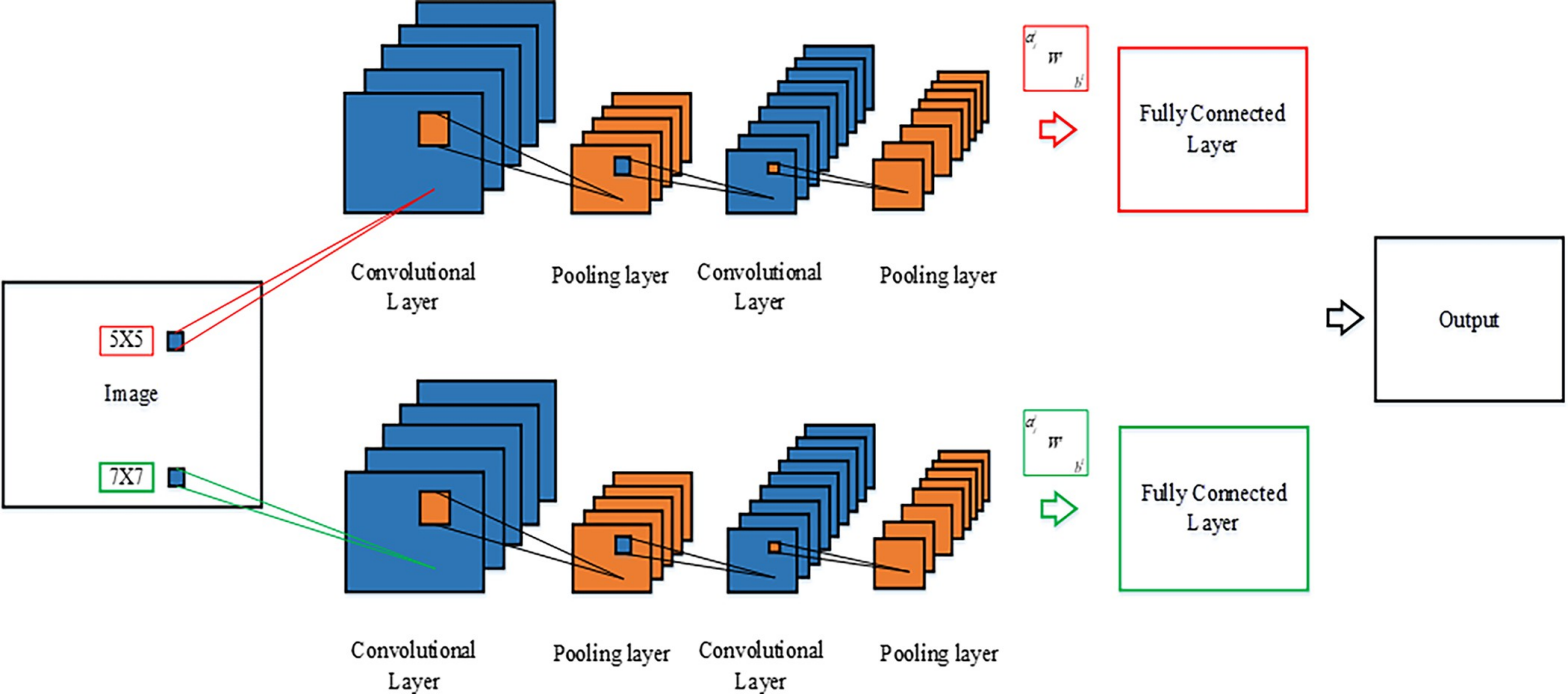
DCCNN structure.

CNN can extract the required features after training. In general, CNN has multiple convolutional and pooling layers. There are many convolution kernels in the convolutional layer to extract local features, and each convolution kernel can map a featured image. Under the same conditions, a small convolution kernel can increase the feature information, reduce the number of parameters, and improve the recognition effect. Therefore, the recognition effect of multiple small convolution kernels is higher than that of large convolution kernels. If the convolution kernels are too small, image features may not be extracted. Traditional CNN uses a fixed-size convolution kernel, so the image granularity is also fixed. Thereby, some features will be lost during the learning process, thus reducing the accuracy of network recognition. DCCNN with dual size convolution kernel has two convolution layers, two pooling layers, and one FC layer, as plotted in Fig 5. The FC layers of the two cellular neural networks are combined using FC mapping and are finally input to a classifier for feature classification.

### Gesture command recognition network

Dynamic gestures contain more information about motion than static gestures. Therefore, how to recognize the gesture is the focus of this section. The optical flow characteristics of the video are analyzed, and the motion information of the video is identified by DCCNN. Video information is divided into spatial information and temporal information. Spatial information refers to the surface information of video frames, while temporal information refers to the optical flow between frames. Thus, spatial and temporal information is extracted from RGB and optical flow images, and DCCNN is applied to identify motion information. The two images are input into the network, respectively, and the predicted results of each network are combined to form the final recognition result. DCCNN consists of two dimensions, space and time, respectively processing spatial and temporal information. One is single-frame CNN, where videos are input via single-frame RGB images to describe the spatial characteristics of the images; Another CNN inputs the video through dense optical streams of multiple frames to describe the temporal properties of the video, combining the two networks to identify the video. DCCNN can acquire motion information of video data effectively. CNN is used to convolve, merge and fully connect spatial feature images and temporal images, and recognize video by combining spatial and temporal features.

**Fig 6:**
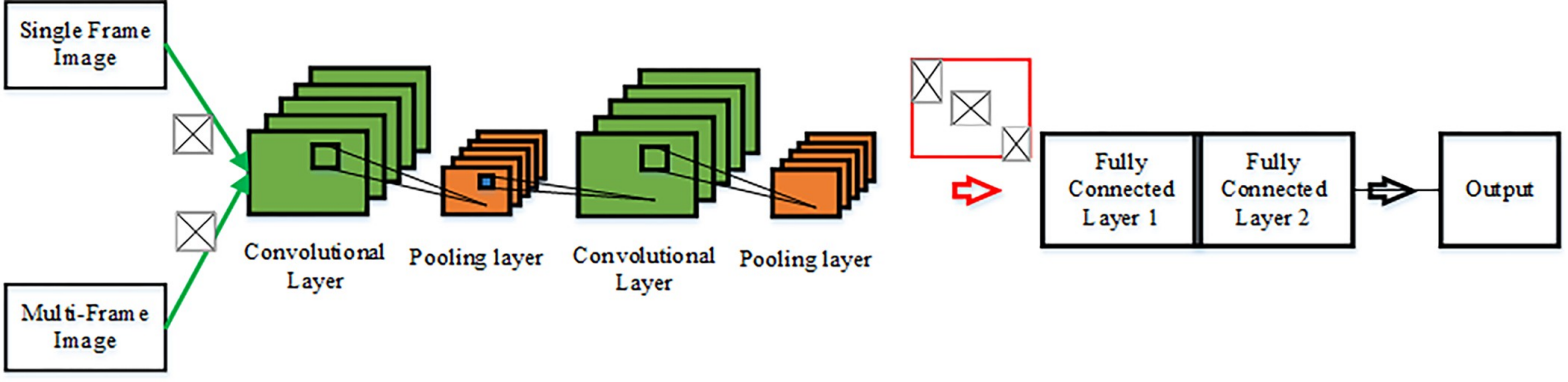
Early merger strategy adopted by DCCNN.

The constructed DCCNN structure uses a convolution kernel in the convolutional layer to convolve single-frame images and multi-frame optical flow images. Its structure is portrayed in Fig 6. The final result is obtained by the weighted sum of the output.

## Results and analysis

### Static gesture command recognition result

**Fig 7:**
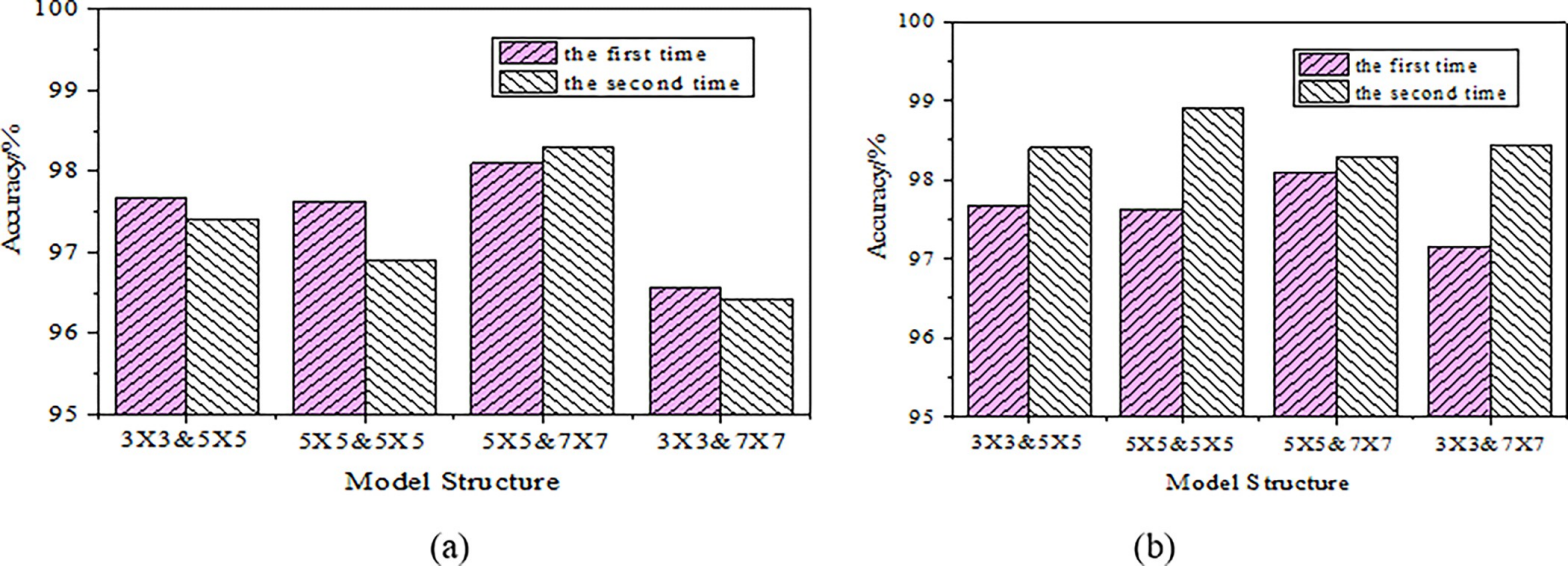
Recognition accuracy of cellular neural networks with different kernel sizes (a.lr = 0.01; b.lr = 0.001).

To verify the influence of the size of the DCCNN kernel on the recognition effect, two parallel experiments are conducted using convolution kernels of different sizes, and the results are denoted in Fig 7.

Fig 7 signifies that the recognition accuracy of DCCNN can be as high as 96%, and the recognition accuracy varies with different convolution kernels. By comparison, it is found that the recognition effect of DCCNN is affected by the size of the convolution kernel. Combining convolution kernels of size 5×5 and 7×7 can improve the recognition accuracy to 98%. The experiment also shows that DCCNN can recognize static gesture images of different scales, and combining the information of these images can obtain richer feature information and better recognition results.

**Fig 8:**
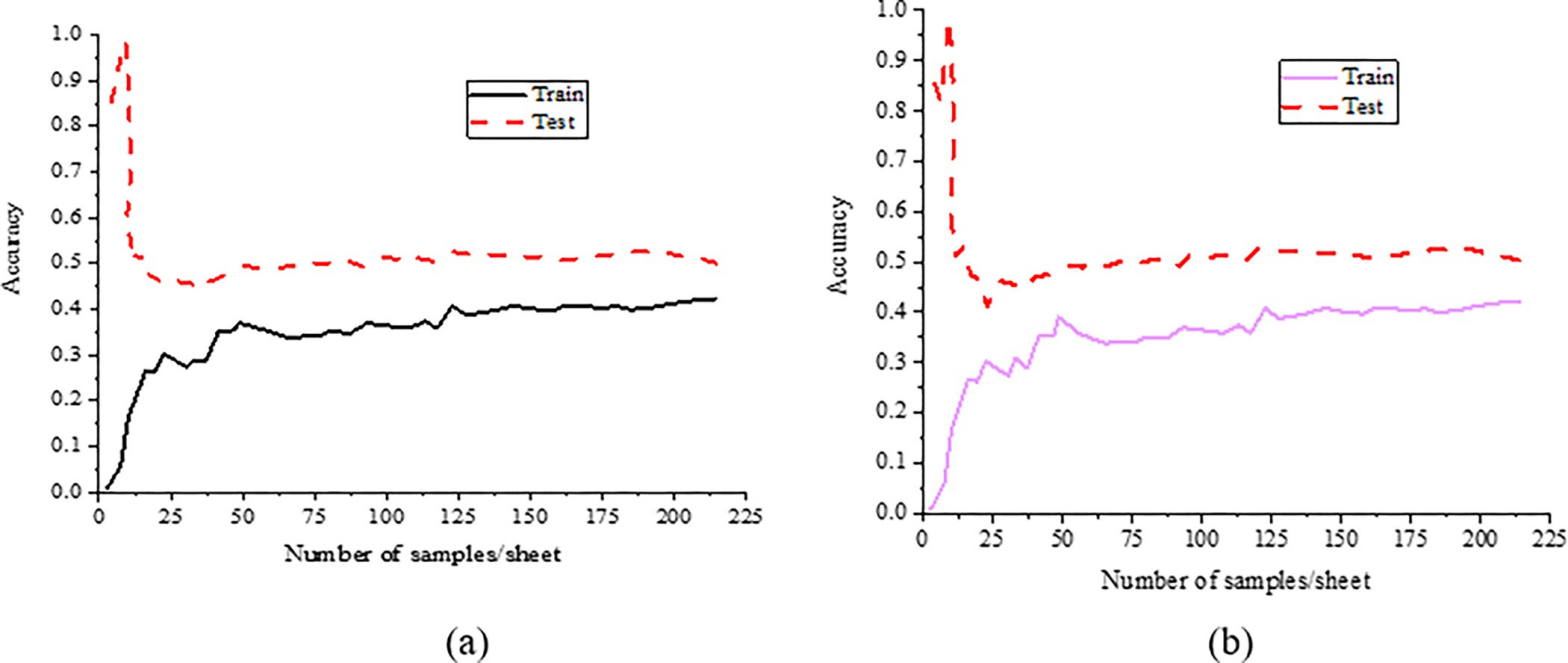
The learning curve (a.lr = 0.01; b.lr = 0.001).

The learning curve of the neural network is suggested in Fig 8.

The learning curve is to display the performance of the network against new data by calculating the accuracy and cross-validation of the training set under different training set sizes, to determine whether the variance or deviation of the network is too high and whether increasing the training set can reduce the overfitting phenomenon of CNN. Fig 8 indicates that there is no overfitting phenomenon in this algorithm.

### Dynamic gesture command recognition result

**Fig 9:**
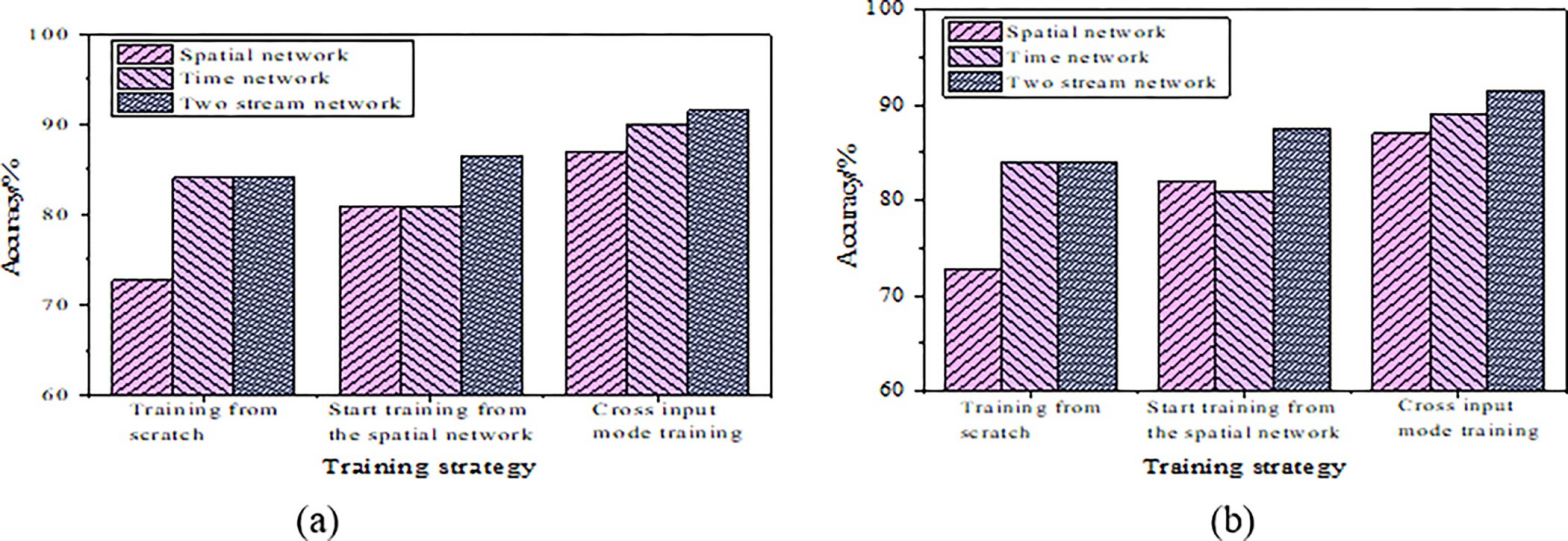
CNN test results under three different training methods (a.lr = 0.01; b.lr = 0.001).

The three types of cellular neural networks involved were tested using training from scratch, pre-training spatial network, and cross-input pre-training strategies, and the results are illustrated in Fig 9.

Fig 9 expresses that both spatial CNN and temporal CNN have universal effects in dynamic gesture recognition, and the accuracy rate is lower than 90%. The accuracy rate of DCCNN is about 90%, which is higher than the other two networks. In addition, if the same CNN is trained using different algorithms, the recognition results are variable. Among them, the recognition effect of cross-input pre-training is the best among the three, and the recognition accuracy is 91%. The reason may be that this training way can effectively reduce the overfitting phenomenon of CNN.

### Test results of system pressure

According to the actual demand, the constructed music teaching system based on the VR system has been subjected to two stress tests, and the test results are exhibited in Table 4.

**Table 4:** Test results of system pressure.

Number of parallel tasks in the system (PCS)	10	20	40	60	80	100	120	150	200
Response time of the first test system(s)	1.6	1.9	1.9	2.2	3.1	3.6	3.8	4	5.5
Response time of the second test system(s)	1.8	2.1	2.1	2.1	3.3	4.1	4.3	4.5	6

As can be seen from the experimental results of the system pressure test in Table 4, when there are 10, 100, and 200 parallel tasks in the system, the response time of the system in the first experiment is 1.6s, and 3.6s, 5.5s, and the response time of the system in the second experiment is 1.8s,4.1s, and 6s. From the overall trend of data change, when the number of test tasks reaches the peak, the system’s response time is 6s, and no abnormal problems occur. It can be found that the designed system has an excellent anti-pressure ability.

The results of the system comparison test are outlined in Table 5.

**Table 5:** Test results for the smoothness of video playback.

Number of video playback devices (PCS)	10	20	40	60	80	100	120	150	200
The playback fluency of the designed system (%)	90	91	92	90	92	90	94	92	91
The playback fluency of the original system (%)	87	80	81	89	80	82	86	85	81

In Table 5, when the number of video playback devices is 10 and 100, the playback fluency of the teaching video of the previous music teaching system is 87% and 82%, while that of the constructed VR-based music teaching system is 90% and 90%. From the overall change trend of the data, it can be seen that with the increasing number of video playback devices, the fluency of system video playback will fluctuate to a certain extent, but the overall trend is declining. Compared with the previous music teaching system, the fluency of teaching video playback of the VR-based music teaching system constructed here has been slightly improved. Thereupon, it means that the proposed system is more suitable for the daily teaching work of colleges and universities.

## Discussion

**Fig 10:**
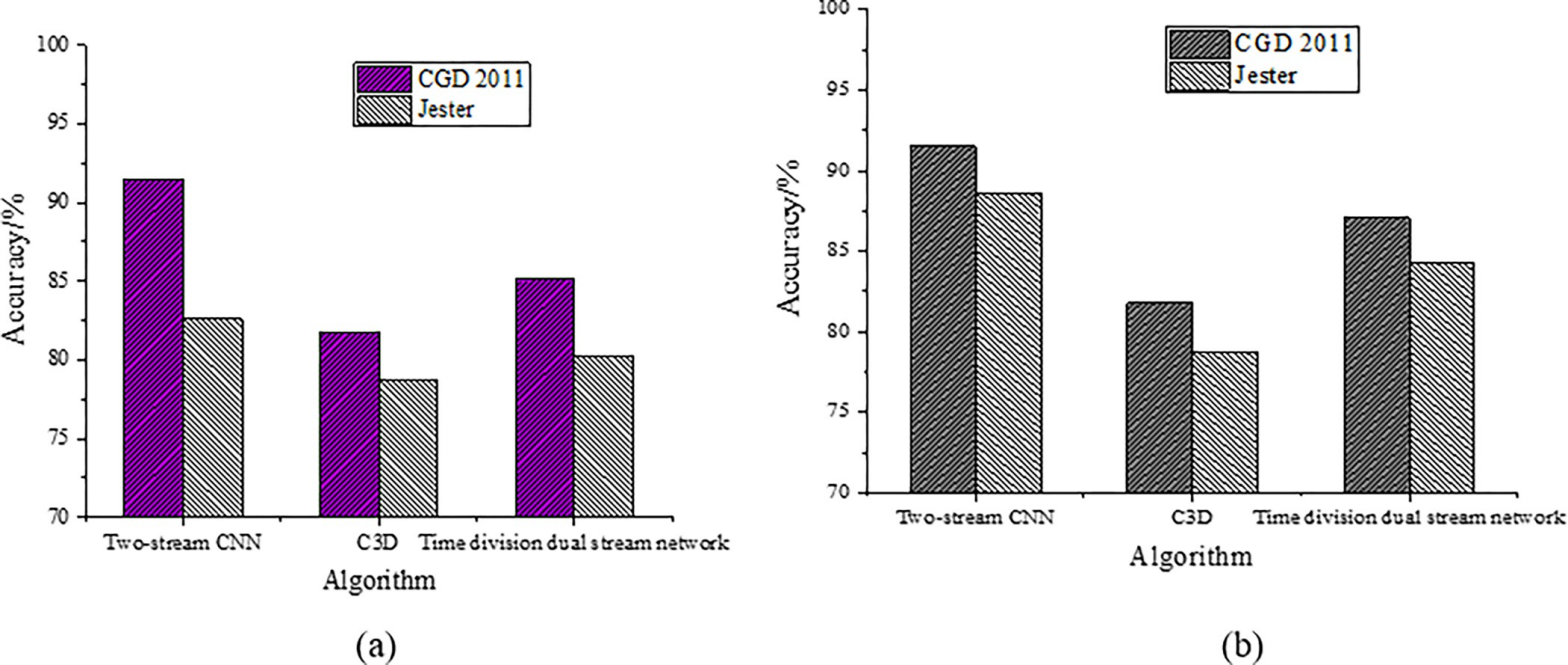
Dynamic gesture recognition results (a.lr = 0.01; b.lr = 0.001).

This study uses two data sets from ChaLearn Gesture Dataset (CGD)2011 and Jester for training. The recognition accuracy of the two-stream CNN proposed by Solanki and Pandey (2019) [34], the two-stream 3D Convolution (C3D) proposed by Du et al. (2019) [35], and the dual-channel algorithm proposed in this study are compared, as revealed in Fig 10.

Fig 10 details that after training with CGD2011 and Jester data sets, the recognition accuracy of dual-channel is 91% and 88%, respectively, higher than that of C3D and two-stream CNN. Hence, the designed algorithm has a good video recognition effect and can avoid the loss of video information in the process of video recognition.

## Conclusion

With the popularity of modern computers, smartphones, and other smart terminals, VR technology will be widely used and further challenge the traditional music teaching mode. Therefore, the application of VR technology in music teaching will be more extensive, such as rhythm skills training. VR technology can simulate related music equipment and construct related equipment simulation training. This study studies the music teaching system based on a virtual simulation system. A virtual piano is developed with Unity3D and related SteamVR plug-in and Leap Motion plug-in as the software platform and HTC Vive kit and Leap Motion sensor fixed on the helmet as the hardware platform. Based on ML theory, a gesture recognition algorithm is proposed and implemented. Specifically, DCCNN is adopted to collect gesture command data of users, and a dual-size convolution kernel is applied to extract feature information in images and gesture commands in videos. The DCCNN then recognizes it. Future work will focus on the above two aspects. (1) The model’s compatibility is further improved, and the code is optimized. (2) Relevant data will be further collected to construct reasonable datasets to understand the actual situation and optimize the model.

## Supporting information

S1 Data (ZIP)
